# The role of microglia in multiple sclerosis: implications for treatment with Bruton’s tyrosine kinase inhibitors

**DOI:** 10.3389/fimmu.2025.1495529

**Published:** 2025-05-15

**Authors:** Patrick Vermersch, Laura Airas, Thomas Berger, Florian Deisenhammer, Nikolaos Grigoriadis, Hans-Peter Hartung, Melinda Magyari, Veronica Popescu, Carlo Pozzilli, Maura Pugliatti, Bart Van Wijmeersch, Magd Zakaria, Celia Oreja-Guevara

**Affiliations:** ^1^ Univ. Lille, Inserm U1172 LilNCog, CHU Lille, FHU, Precise, Lille, France; ^2^ Division of Clinical Neurosciences, University of Turku, Turku, Finland; ^3^ Neurocenter of Turku University Hospital, Turku, Finland; ^4^ Department of Neurology, Medical University of Vienna, Vienna, Austria; ^5^ Comprehensive Center for Clinical Neurosciences and Mental Health, Medical University of Vienna, Vienna, Austria; ^6^ Department of Neurology, Medical University of Innsbruck, Innsbruck, Austria; ^7^ Laboratory of Experimental Neurology and Neuroimmunology, Second Department of Neurology, AHEPA University Hospital, Aristotle University of Thessaloniki, Thessaloniki, Greece; ^8^ Department of Neurology, Medical Faculty, Heinrich-Heine-University Düsseldorf, Düsseldorf, Germany; ^9^ Brain and Mind Center, University of Sydney, Sydney, NSW, Australia; ^10^ Department of Neurology, Palacky University Olomouc, Olomouc, Czechia; ^11^ Danish Multiple Sclerosis Center, Department of Neurology, Rigshospitalet, Copenhagen University Hospital, Copenhagen, Denmark; ^12^ Department of Clinical Medicine, University of Copenhagen, Copenhagen, Denmark; ^13^ University MS Centre, Hasselt-Pelt, Belgium; ^14^ Revalidatie & Multiple Sclerosis (MS), Noorderhart, Pelt, Belgium; ^15^ Hasselt University Belgium, Hasselt, Belgium; ^16^ Multiple Sclerosis Center, S. Andrea Hospital, Department of Human Neuroscience, University Sapienza, Rome, Italy; ^17^ Department of Neuroscience and Rehabilitation, University of Ferrara, Ferrara, Italy; ^18^ UNIFE, Interdepartmental Center of Research for Multiple Sclerosis and Neuro-inflammatory and Degenerative Diseases, University of Ferrara, Ferrara, Italy; ^19^ Department of Neurology, Ain Shams University, Cairo, Egypt; ^20^ Department of Neurology, Hospital Clinico San Carlos, IdISSC, Madrid, Spain; ^21^ Departamento de Medicina, Facultad de Medicina, Universidad Complutense de Madrid (UCM), Madrid, Spain

**Keywords:** central nervous system, disease management, microglia, multiple sclerosis, neuroinflammation

## Abstract

**Background:**

Multiple sclerosis (MS) is a chronic autoimmune disease affecting the central nervous system (CNS), characterized by inflammation and neurodegeneration. The pathophysiology of MS, especially its progressive forms, involves various cellular components, including microglia, the primary resident immune cells of the CNS. This review discusses the role of microglia in neuroinflammation, tissue repair, and neural homeostasis, as well as their involvement in MS and explores potential therapeutic strategies targeting microglial function.

**Methods:**

A literature search conducted in August 2023 and updated in March 2025, using the PubMed database, focused on articles relating to microglia and MS published in 2018–2025. Additionally, ongoing clinical trials of Bruton’s tyrosine kinase (BTK) inhibitors were identified through the ClinicalTrials.gov website in November 2023 and updated in March 2025.

**Results:**

Microglia are highly adaptive and exhibit various functional states throughout different life stages and play critical roles in neuroinflammation, tissue repair, and neural homeostasis. Their altered activity is a prominent feature of MS, contributing to its pathogenesis. Imaging techniques such as magnetic resonance imaging (MRI) and positron emission tomography (PET) provide insights into microglial activity in MS. BTK inhibitors and other novel treatments for MS, including masitinib and frexalimab, show promise in modulating microglial function and influencing the disease progression rate.

**Conclusions:**

The multifaceted roles of microglia in CNS development, immune surveillance, and particularly in the pathogenesis of MS highlight the potential of targeting microglial functions in MS treatment. Emerging research on the involvement of microglia in MS pathophysiology offers promising avenues for developing novel therapies, especially for progressive MS, potentially improving patient outcomes in this debilitating disease.

## 1 Introduction

Multiple sclerosis (MS) is a chronic autoimmune disease of the central nervous system (CNS) characterized by multifocal inflammatory processes, demyelination, and axonal loss affecting both white and grey matter (1–3). CNS damage occurs over time, such that the advanced disease stages are characterized by irreversible disability (3). Worldwide, approximately 2.8 million people were estimated to have MS in 2020, a 30% increase compared with 2013 (4). MS typically manifests in young adults and is recognized as one of the leading causes of non-traumatic neurological disability in this age group (4, 5). MS imposes a substantial socioeconomic and personal burden as a result of direct and indirect costs, reduced quality of life, and significant challenges in daily functioning (4).

Patients with relapsing-remitting MS (RRMS) have a disease course consisting of periods of stable neurologic disability between relapses, while those with progressive MS have an increasing level of neurologic disability regardless of the occurrence of relapses (6). Primary progressive MS (PPMS) is characterized by a progressive course from disease onset, while secondary progressive MS (SPMS) is defined as an initial relapsing-remitting disease course followed by progressive disease (6). While there are effective treatments for relapsing MS (RMS), there are limited therapeutic options for progressive MS, including both SPMS and PPMS (7). This reflects the spectrum of pathophysiological mechanisms that extend and intensify from relapsing-remitting MS (RRMS) to progressive forms of MS, including an exacerbation of chronic inflammation behind the blood-brain barrier (7).

Various cellular components play a central role in the pathophysiology of progressive MS (8, 9). Microglia have gained increasing attention, particularly for their involvement in the development of progressive forms of MS. As the primary resident immune cells of the CNS, microglia play a pivotal role in neuroinflammation, tissue repair, and neural homeostasis, and their activity is altered in MS (7–9). Microglia also play a crucial role in neurodegenerative diseases. They serve as the first line of immune defense and function in synapse pruning, injury repair, homeostasis maintenance, and regulation of brain development through scavenging and phagocytosis. In neurodegenerative diseases such as Alzheimer’s disease, Parkinson’s disease, and amyotrophic lateral sclerosis, microglia increase their rates of proliferation and are associated with the phagocytosis and clearance of pathological proteins, which, if impaired, can lead to neuroinflammation and eventually neurodegeneration. Microglia can both protect and harm neurons. They promote axonal regeneration, remyelination, clearance of inhibitory myelin debris, and the release of growth factors. However, in response to specific stimuli or with neuroinflammation, microglia also have the capacity to damage and kill neurons.

Although there are several treatments available for MS, many are associated with risks, including increased rates of infection. Thus, there are still unmet treatment needs for patients with MS, including lower risk, effective immunotherapies, neuroprotective therapies, and therapies that result in neurological repair and functional improvement (10). Inhibition of Bruton’s tyrosine kinase (BTK) has been shown to alter B cell activation, decrease phagocytosis, prevent microglial activation, and reduce the secretion of pro-inflammatory cytokines, and the inhibition of microglial activation may promote remyelination (10); since BTK inhibitors may act on both B cells and microglia, use of BTK inhibitors may have benefits in both RMS and progressive MS (10). This review aims to outline the basic characteristics of microglia, discuss their involvement in MS pathology, and explore the potential of BTK inhibitors in MS management.

## 2 Methods

A search of the literature was conducted in August 2023 and updated in March 2025 using the PubMed database and the following terms: (“microglia”[MeSH] OR “microglia”[Title/Abstract]) AND (“multiple sclerosis”[MeSH] OR “multiple sclerosis”[Title/Abstract]). The search was limited to English-language articles published in 2018–2025. Publications identified from the reference lists and other publications known to be relevant were also cited.

A search of the ClinicalTrials.gov website was conducted in November 2023 to identify ongoing clinical trials of BTK inhibitors, and updated in March 2025.

## 3 Basic background on microglia

Glia are the non-neuronal cells of the nervous system, represented in the adult human brain by astrocytes, oligodendrocytes, and microglia (11, 12). Microglia are specialized, resident macrophages of the CNS (11). They are relatively small (7–10 µm) mononuclear macrophages characterized by variable morphology (9, 13). Microglia account for approximately 10% of cells in the adult human brain; however, their density varies from 5–12% depending on the region (11, 14). Microglial cells form the largest population of mononuclear macrophages within the CNS and are distributed throughout the parenchyma, where they are found in close proximity to neurons (8, 13).

A variety of appearance types have been described; however, two highly distinct shapes are commonly mentioned: ameboid and ramified (9, 14–16). Ramified microglia have a small body and long, branching processes. This morphology is characteristic of the homeostatic state when microglia perform their immunological surveillance functions. Ameboid microglia have a relatively large body and few or no processes. This form is actively phagocytic and is thought to represent the principal morphological phenotype of activated microglial cells. Ameboid microglia are also prevalent during brain development. Microglia exhibit significant morphological variation both within and between CNS regions (17), and between white and gray matter lesions in patients with MS (described below) (18).

Microglia differ from the infiltrating peripheral macrophages in a number of ways (14). Importantly, microglia originate from the yolk sac during early embryonic development, whereas peripheral macrophages originate from hematopoietic stem cells in the bone marrow (19). Although microglia and peripheral macrophages express many of the same cell surface markers, including CD11b/c, F4/80, CX3CR1, CD45 and IBA-1, there are specific differences (14). For example, microglia express Siglec-H, which is largely absent from the surface of infiltrating peripheral macrophages. On the other hand, peripheral macrophages exhibit CD44 and CD169, neither of which are expressed by microglia. Mouse CD33-related Siglec-H is a microglia-specific marker that plays an important role in modulating leukocyte behavior by suppressing toll-like receptor 9 (TLR9)-induced interferon-α production in plasmacytoid dendritic cells (20), and appears to control TLR9-dependent post-viral inflammatory responses, as well as regulate chemokine responsiveness of plasmacytoid dendritic cells (21). CD44 is an activation marker for glomerular parietal epithelial cells (PECs), and CD44-overexpressing glomerular PECs promote glomerular scarring in experimental focal segmental glomerulosclerosis (FSGS) (22). CD169-positive macrophages play important functions in immune regulation and several human diseases, and can serve as an effective indicator of disease progression and prognosis (23).

## 4 Role of microglia in health and disease

**Figure 1:**
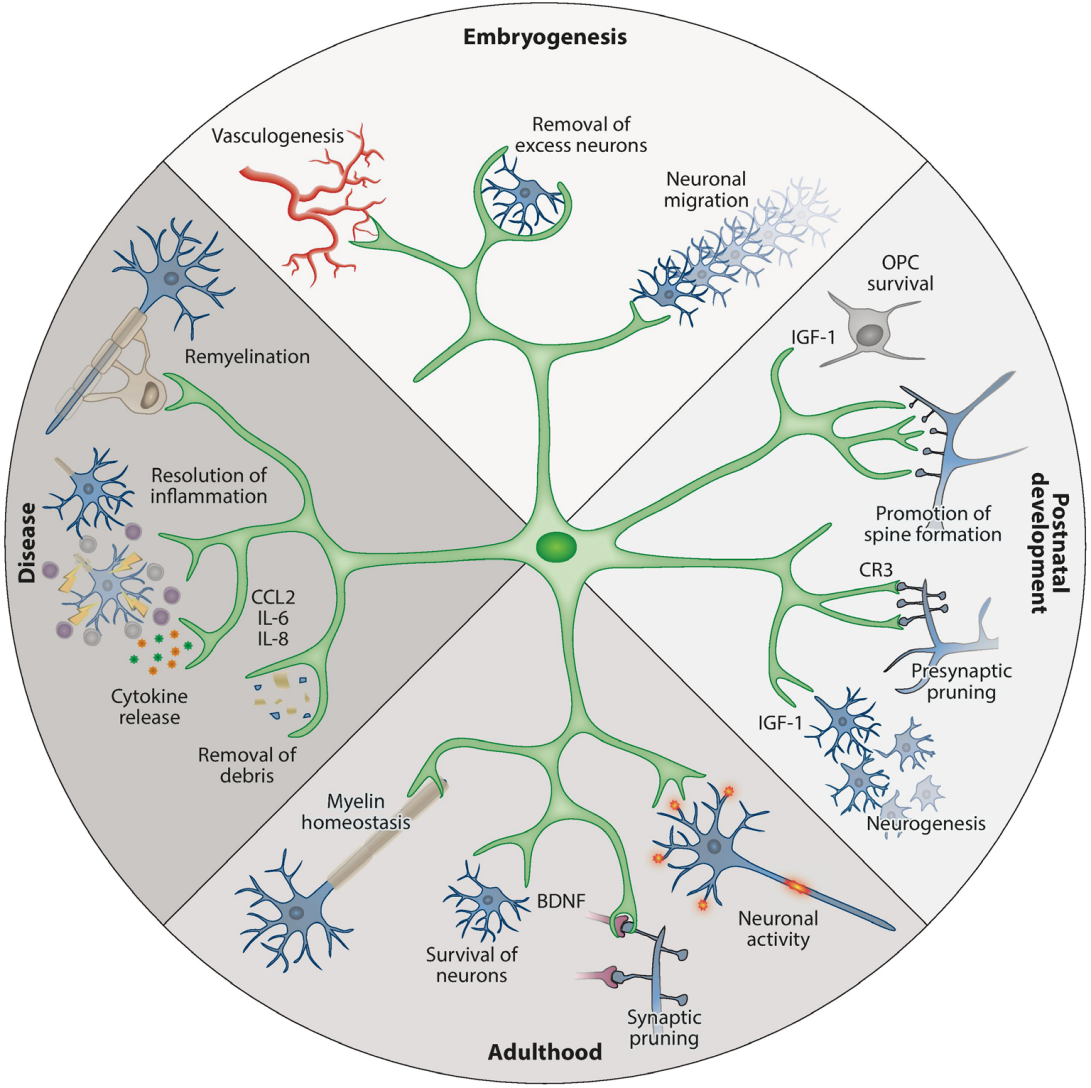
An overview of the physiological functions of microglia during development and adulthood (13). (A) Top: during embryogenesis, microglia promote vessel development, remove superfluous neurons, and guide neuronal migration. (B) Right: at postnatal time points, microglia support OPC survival and development (e.g., by IGF-1 production), support neurogenesis in defined brain areas, and promote neuronal spine formation. (C) Bottom: in adulthood, their functions shift to ensure the survival of neurons (e.g., by BDNF production) and oligodendrocytes. (D) Left: in response to infection, injury, or dysfunction, microglia are activated and participate in phagocytosis and the production of soluble factors such as cytokines, chemokines, and surface markers. BDNF, brain-derived neurotrophic factor; CCL2, C-C motif chemokine ligand 2; CR3, complement receptor 3; IGF-1, insulin-like growth factor 1; IL, interleukin; OPC, oligodendrocyte precursor cell. Used with permission of Annual Reviews, Inc., from Prinz M et al. Annu Rev Immunol (2021) 39:251-77 (doi: 10.1146/annurev-immunol-093019-110159); permission conveyed through Copyright Clearance Center, Inc.

Microglial cells perform a wide variety of functions, which demands a high degree of plasticity and adaptability (**Figure 1**) (13, 24, 25).

Gene expression studies have shown that microglial cells have distinct profiles during the embryonic, early post-natal, and adult stages (26). During embryonic development, microglia display amoeboid morphology, have high proliferation rates, and are actively engaged in phagocytosis (24). As the brain transitions into postnatal steadiness, a shift is observed from the expansive phase of microglia to a stage marked by modest homeostatic proliferation.

Microglial cells originate in the yolk sac and migrate to the developing brain at approximately the same time as the development of neurons is taking place, and before the appearance of other types of glia (13, 24). Microglia are intricately involved in the development of the neuronal architecture of the embryonic brain by phagocytosing excess or apoptotic neuronal progenitor cells (NPCs), while supporting the survival, proliferation, migration, and maturation of other NPCs and neurons (**Figure 1** Top) (13, 24). In addition, microglia support the formation and elimination of synapses and promote the development of brain vasculature (8, 11, 14).

In the neonatal period, microglia remain vital to healthy brain development by facilitating myelinization through interactions with oligodendrocytes and their progenitors, thereby supporting the survival of layer V neurons in the somatosensory cortex, and by engaging in synaptic pruning (**Figure 1** Right) (13, 24).

In the adult brain, microglia are the dominant immune cells and play critical roles in maintaining CNS homeostasis (**Figure 1** Bottom) (14). Microglia are engaged in continuous immunological surveillance by extending and retracting their processes (24). Upon detecting signs of infection, injury, or dysfunction, microglia become activated and transition from the homeostatic ramified form into proliferating amoeboid microglia (**Figure 1** Left) (14). Activation signals can be exogenous molecules such as pathogen-associated molecular patterns (PAMP), bacterial lipopolysaccharides (LPS), or foreign genetic material, or endogenous signals such as danger/damage-associated molecular patterns (DAMP), amyloid-β senile plaques, or cytokines released by other microglial cells and astrocytes.

The M1/M2 paradigm, originally developed to describe activation states in T helper (Th) cells and peripheral macrophages, has been applied to microglia (14). In this paradigm, M1 describes a pro-inflammatory activation state that directs microglia to neutralize threats and is adopted in response to bacterial-derived LPS (14), interferon-γ released by Th cells (14) and tumor necrosis factor-alpha (TNF-α) produced by other pro-inflammatory activated microglia (27, 28). In addition to TNF-α, M1 microglia produce and release other pro-inflammatory cytokines, such as interleukin (IL)-1β, IL-6, IL-12, IL-17, IL-18 and IL-23, and these pro-inflammatory cytokines play a role in the maintenance of inflammation (14). Activated M1 microglia also secrete chemokines such as CCL5, CCL20, CXCL1, CXCL9, and CXCL10, which act to recruit immune cells, and matrix metalloproteinase (MMP)-12 and MMP-9; MMP-9 perpetuates a pro-inflammatory state by promoting IL-1β maturation (14). As part of mounting a defense against the foreign pathogen, activated M1 cells produce inducible nitric oxide synthase (iNOS), translocator protein (TSPO), and reactive oxygen species (14, 29); they also participate in antigen presentation by utilizing major histocompatibility complex II (14). M2 is an anti-inflammatory alternative activation state associated with wound healing, tissue repair, and resolution of inflammation. M2 activation is induced by the presence of IL-4, IL-10 and IL-13, which are secreted by microglia (14), and this microglial state is designed to deactivate pro-inflammatory cell phenotypes and restore homeostasis (14, 29). In addition to IL-4, IL-10 and IL-13, M2 microglia produce and release anti-inflammatory and tissue repair factors, including transforming growth factor-beta, the chemokines CCL2, CCL17, CCL22, and CCL24, growth factors such as insulin-like growth factor I and fibroblast growth factor, colony-stimulating factor 1, as well as the neurotrophic factors nerve growth factor, brain-derived neurotrophic factor, neurotrophins 4/5, and glial cell–derived neurotrophic factor, and the pro-survival factor progranulin (14).

However, it is crucial to note that the M1/M2 paradigm is an oversimplification and is considered to be obsolete, as in reality, microglial activation occurs across a continuous spectrum, with cells exhibiting a variety of phenotypes positioned between these two poles (14, 30). A wide variety of microglia activation states have been described; however, the nomenclature used to denote them is in a state of flux because this field is still evolving (30). In general, homeostatic microglia are characterized by the expression of P2RY12 and TMEM119, which are downregulated in reactive states (31). Interestingly, levels of P2RY12 and TMEM119 expression can apparently change in human white- and gray matter-derived microglia exposed to inflammatory mediators such as interferon-γ and LPS.

The metabolic state of microglia is intrinsically linked to their function, and several pathways and key molecules are involved in homeostasis and in response to (**Table 1**) (32, 33). The main function of homeostatic microglia is to survey the brain parenchyma to maintain brain homeostasis (34), with key nutrients such as glucose, glutamine and fatty acids involved in supporting the function of homeostatic microglia (32). In homeostasis, microglia mainly generate energy via oxidative metabolism, with key pathways including glycolysis, the tricarboxylic acid (TCA) cycle and oxidative phosphorylation, but other pathways, such as those involving fatty acids, also contribute (**Table 1**) (32, 33). Under pro-inflammatory conditions, microglia metabolism shifts from the oxidative phosphorylation of homeostasis to aerobic glycolysis, also increasing metabolism via increases in the pentose phosphate pathway (PPP) and fatty acid synthesis (**Table 2**) (32, 33). Reactive oxygen species generated by the PPP play a role in the modulation of microglia phagocytosis, activation of the mitogen-activated protein kinase pathway, and immunoreactions in microglia and macrophages, while fatty acid synthesis results in secretion of pro-inflammatory cytokines (33). Under anti-inflammatory conditions, microglia generate energy via oxidative metabolism, similar to homeostatic microglia (33). Under these conditions, oxidative phosphorylation, the TCA cycle, and fatty acid oxidation are increased, while glycolysis, fatty acid synthesis and the PPP are decreased (**Table 1**) (32, 33). The use of oxidative metabolism allows the microglia to maintain their neuroprotective functions and contributes to tissue repair and wound healing in the longer term; the production of ornithine by anti-inflammatory microglia as part of glutaminolysis enhances cell proliferation and repair (33).

**Table 1:** Key pathways and molecules involved in microglia metabolism under homeostatic and inflammatory conditions (32, 33).

Cycles/pathways and molecules involved in microglia homeostasis	TCA cycle NADH FADH_2_ ↓ OX PHOS	OX PHOS Electron transport chain ↓ ATP Mitochondrial ROS	Glycolysis Glucose ↓ Pyruvate ↓ Acetyl-CoA ↓ TCA cycle ↓ OX PHOS ↓ ATP	PPP Glucose ↓ Ribose-5-phosphate ↓ NADPH ↓ Elongated FAs ROS	Glutaminolysis Glutamine ↓ Glutamate ↓ TCA cycle ↓ OXPHOS	FA oxidation Fatty acyl-CoA ↓ Acetyl-CoA ↓ TCA cycle ↓ OX PHOS ↓ ATP	FA synthesis Acetyl-CoA ↓ Malonyl-CoA ↓ Elongated FAs
Impact of pro-inflammatory stimulation	Cycles/pathways increased: glycolysis, glutaminolysis, PPP, FA synthesis Cycles/pathways decreased: OX PHOS, TCA cycle, FA oxidation						
Impact of anti-inflammatory stimulation	Cycles/pathways increased: OX PHOS, TCA cycle, FA oxidation Cycles/pathways decreased: glycolysis, FA synthesis, PPP						

ATP, adenosine triphosphate; CoA, coenzyme A; FA, fatty acid; FADH_2_, Flavin adenine dinucleotide; NADH, nicotinamide adenine dinucleotide; OX PHOS, oxidative phosphorylation; PPP, pentose phosphate pathway; ROS, reactive oxygen species; TCA, tricarboxylic acid.

**Table 2:** Recent and ongoing clinical trials of selected investigational agents targeting microglia in MS.

Study	Design	Phase	Comparator	Indication	N^a^	Estimated completion date
*Tolebrutinib*						
NCT04742400	Non-R, PG, OL	2	N/A	MS	12	December 2025
NCT03996291	SG, OL	2	N/A	RMS	125	November 2024^b^
HERCULES, NCT04411641	R, PG, TB	3	Placebo	SPMS	1131	August 2024^b^
PERSEUS, NCT04458051	R, PG, TB	3	Placebo	PPMS	767	November 2025
GEMINI 1, NCT04410978	R, PG, TB	3	Teriflunomide	RMS	974	July 2024^b^
GEMINI 2, NCT04410991	R, PG, TB	3	Teriflunomide	RMS	899	July 2024^b^
*Fenebrutinib*						
FENopta, NCT05119569	R, PG, DB	2	Placebo	RMS	109	December 2026
FENhance, NCT04586010	R, PG, DB	3	Teriflunomide	RMS	746	November 2025
FENhance 2, NCT04586023	R, PG, DB	3	Teriflunomide	RMS	751	November 2025
FENtrepid, NCT04544449	R, PG, DB	3	Ocrelizumab	PPMS	985	December 2026
*Evobrutinib*						
NCT02975349	R, PG, DB	2	Placebo, DMF	RRMS	267	April 2024^b^
evolutionRMS 1, NCT04338022	R, PG, QB	3	Teriflunomide	RMS	1124	March 2024^b^
evolutionRMS 2, NCT04338061	R, PG, QB	3	Teriflunomide	RMS	1166	March 2024^b^
*Remibrutinib*						
REMODEL-2, NCT05156281	R, PG, DB	3	Teriflunomide	RMS	800^c^	October 2030
NCT05147220	R, PG, DB	3	Teriflunomide	RMS	800^c^	October 2030
*Orelabrutinib*						
NCT04711148	R, PG, QB	2	N/A	RRMS	160^c^	March 2026
Masitinib						
MAXIMS, NCT05441488	R, PG, QB	3	Placebo	PPMS or SPMS	800^c^	December 2025
*Frexalimab*						
NCT06141473	R, PG, QB	3	Teriflunomide	RMS	1400^c^	May 2027
NCT06141486	R, PG, QB	3	Placebo	SPMS	900^c^	March 2028
*Vidofludimus*						
ENSURE-1, NCT05134441	R, PG, QB	3	Placebo	RMS	1050^c^	September 2032
ENSURE-2, NCT05201638	R, PG, QB	3	Placebo	RMS	1050^c^	October 2032

a Actual number of patients enrolled, unless indicated otherwise.

b Actual study completion date.

c Estimated number of patients enrolled.

BTK, Bruton’s tyrosine kinase; DB, double-blind; DMF, dimethyl fumarate; MS, multiple sclerosis; N/A, not available; OL, open-label; PG, parallel-group; PPMS, primary progressive multiple sclerosis; QB, quadruple-blind; R, randomized; RMS, relapsing multiple sclerosis; RRMS, relapsing-remitting multiple sclerosis; SG, single-group; SPMS, secondary progressive multiple sclerosis; TB, triple-blind.

## 5 Microglia in the context of multiple sclerosis

**Figure 2:**
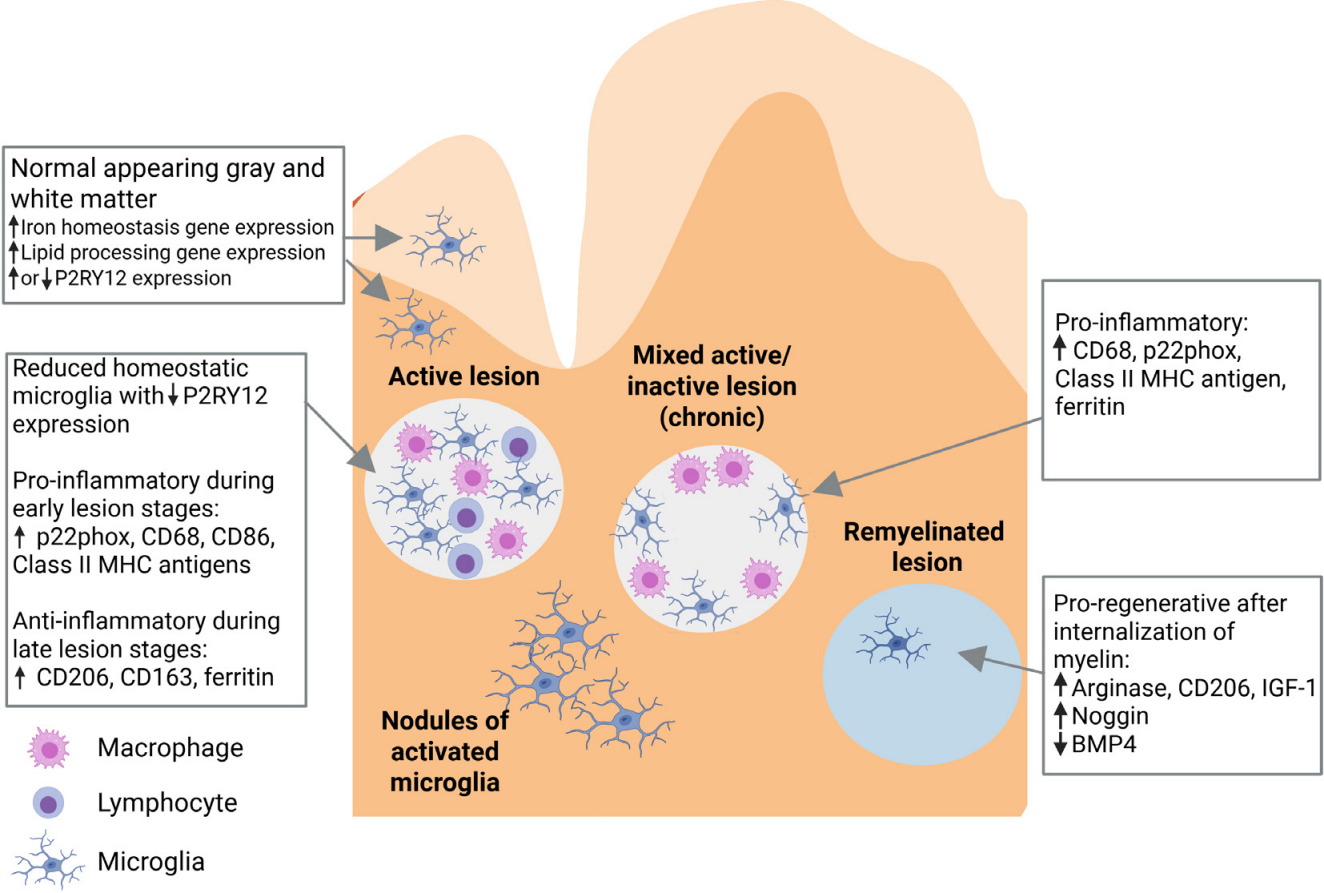
Role of microglia in the pathophysiology of multiple sclerosis (16). Pathological changes in multiple sclerosis include the presence of variable microglia phenotypes in normal-appearing white matter and normal-appearing gray matter, and in active, mixed active/inactive, and inactive (remyelinated) white matter lesions. BMP4, bone morphogenetic protein 4; CD, cluster of differentiation; IGF-1, insulin-like growth factor 1; MHC, major histocompatibility complex. Figure adapted from Guerrero BL et al. *Front Immunol* (2020) 11:374 (doi: 10.3389/fimmu.2020.00374), under a CC BY 4.0 DEED license (creativecommons.org.licenses/by/4.0), where the changes made were exclusively to the figure legend (the legend text was slightly revised, further text was added for explanation, and a list of abbreviations was added).

As the central immune effector cells of the CNS, microglia play a pivotal role in the pathophysiology of MS (**Figure 2**) (16). In degenerative diseases such as MS, microglia become dysfunctional, which is a state associated with damaged mitochondria, increased glycolysis, release of pro-inflammatory cytokines and reactive oxygen species, lipid metabolism deficits, and loss of migratory and phagocytic functions (33, 34). The innate inflammatory responses of microglia are the drivers of the sustained, detrimental CNS inflammation seen in MS (32). A subset of microglia has been defined as “microglia inflamed in MS” (MIMS) that are characterized by neurodegenerative programming, where the transcriptional programming overlaps with disease-associated microglia (35). Two subsets have been described: MIMS-foamy, which have an expression profile related to foam-cell differentiation, lipid storage, response to lipoprotein particles, and regulation of inflammatory response, suggesting that these cells are involved in myelin phagocytosis and clearance (35); and MIMS-iron, which have an expression profile suggesting a role in antigen presentation and direct inflammatory damage at the edge of lesions (35). T cells, plasma blasts/plasma cells and MIMS themselves are involved in regulation of MIMS target genes (35).

A characteristic neuropathological finding in MS is the presence of focal demyelinating white matter lesions, which are often located around a central vein (36–38). These lesions are characterized by a leaky blood-brain barrier, immune cell infiltration, plasma protein deposition, breakdown of myelin, loss of oligodendrocytes, and varying degrees of axonal damage. When classified according to stage (from earliest to latest), active, mixed active/inactive, and inactive lesions can be identified. In active lesions, microglia and macrophages are distributed throughout the entire area. Mixed active/inactive lesions are characterized by a hypocellular center, surrounded by a rim of activated microglia and macrophages. In inactive lesions, microglia and macrophages are nearly absent (36–38). In addition, areas of remyelination are known as shadow plaques. Slowly expanding, chronically active, or smoldering lesions are a variety of mixed lesions characterized by continual radial expansion (39). These lesions appear to be more prevalent in PPMS than RRMS and are associated with poor prognosis (39, 40).

Microglial cells within active white matter lesions are characterized by the absence of homeostatic markers such as P2RY12 and an upregulation of CXCR4, indicating a shift to a disease-associated state (31, 41, 42). Microglial cells located at the edges of mixed active/inactive lesions, where the process of demyelination is actively occurring, display an activated morphology, have a pro-inflammatory phenotype, and express iNOS (16, 18, 43). These cells are engaged in the phagocytosis of myelin remnants, dead oligodendrocytes, and extravasated erythrocytes, resulting in the accumulation of iron which can be detected using magnetic resonance imaging (MRI) techniques such as susceptibility-weighted imaging (SWI, see below). Microglial cells located in the interior of active lesions have an anti-inflammatory phenotype.

Microglial cells comprise approximately 40% of the phagocytes found in early active lesions (16, 39). As the lesion develops, the proportion of microglial cells decreases as peripheral macrophages are recruited to the site (16). However, microglia dominate active lesions in progressive MS, as well as smoldering lesions, while monocyte-derived macrophages are more common in the lesions of RRMS (39). A correlation has been found between the presence of pro-inflammatory microglia and early-stage, but not late stage, oligodendrocytes in poorly remyelinating donors, suggesting that pro-inflammatory microglia may inhibit the maturation of oligodendrocyte progenitor cells, leading to remyelination failure (44).

Pathological changes in MS are not confined to focal lesions and can be present throughout normal-appearing white matter (NAWM) and normal-appearing gray matter (NAGM) (43). Nodules of activated microglial cells are found throughout NAWM; however, it is unclear whether these represent early-stage lesions or form as a result of Wallerian degeneration (39, 43). In addition, similar to the MIMS discussed above, microglial cells found in the NAWM of patients with MS exhibit upregulation of the genes involved in lipid processing, while those found in NAGM exhibit upregulation of the genes involved in iron homeostasis (16). Microglia in subpial gray matter lesions display a homeostatic morphology, and express genes associated with the canonical Wnt signaling pathway (18). Microglial activation in NAWM increases with advancing MS disease (45).

Several cerebrospinal fluid (CSF) biomarkers of microglial activation have been identified. For example, the CSF biomarker soluble triggering receptor expressed on myeloid cells 2 (sTREM2) is a marker of microglial activity which may be associated with neuroaxonal damage related to counter-regulation of TREM2, a membrane receptor expressed on microglia and macrophages that promotes debris phagocytosis (46). High levels of sTREM2 have been shown to predict worse MS disease severity in patients with RRMS, as assessed by the MS Severity Score (46). Elevated CSF levels of chitinase-3-like-1 (CHI3L1) have been linked to cognitive impairments in early MS, and the progression to clinically definite MS, as well as disease progression in general; CHI3L1 has been shown to activate microglia and macrophages, thereby indirectly promoting demyelination (47). In terms of blood biomarkers, a recent study found that extracellular vesicles derived from human blood co-expressing UCHL1 and CX3CR1 were potential biomarkers specific to microglia (48).

## 6 Imaging techniques in microglia research

Studying microglia in living patients is crucial for understanding the involvement of these cells in the pathogenesis of MS. Two prominent non-invasive techniques can be used for visualizing microglia *in vivo*: MRI and positron emission tomography (PET).

MRI provides high spatial resolution images of the brain; however, it is not inherently sensitive to microglia (35). Instead, certain MRI techniques, such as SWI, quantitative susceptibility mapping (QSM), and the use of gadolinium-based contrast agents and ultrasmall superparamagnetic iron oxide nanoparticles (USPIOs), can help deduce microglial activity or presence due to their tendency to uptake iron whilst in an activated state (49–51).

Using traditional MRI sequences, such as T2-weighted fast spin-echo and fluid-attenuated inversion recovery (FLAIR), MS lesions are visualized as oval-shaped hyperintense areas (52). However, one drawback of such sequences is their lack of specificity, as T2 hyperintense lesions can indicate a wide variety of pathological processes, including inflammation, demyelination, remyelination, gliosis, edema, Wallerian degeneration, and axonal damage. More advanced MRI techniques, such as SWI and QSM, address this limitation. SWI is an MRI technique that uses phase information derived from gradient-echo imaging with relatively long echo times to improve contrast (53). QSM is a post-processing technique that uses the phase data to calculate and map the magnetic susceptibility values (54). Researchers have also begun investigating diffusion-weighted MRI; in rat models, diffusion-weighted MRI was able to detect microglia and astrocyte activation in the setting of neuroinflammation, degeneration, and demyelination, with the specific population signatures able to be non-invasively quantified (55).

The significance of iron in the context of MRI comes from its paramagnetic properties. In normal brain tissue, iron is found primarily in oligodendrocytes, where it plays a crucial role as a cofactor in the synthesis of myelin (56). On the other hand, in the brain tissue of patients with MS, iron accumulates within microglia and macrophages that phagocytose myelin fragments, dead oligodendrocytes, and extravasated erythrocytes. In these cells, iron induces a pro-inflammatory activation state (57). Iron accumulation also occurs in T lymphocytes, influencing their differentiation and pathogenicity. As a result, chronic active lesions appear to have a characteristic paramagnetic rim on SWI (35, 56). Studies combining the use of QSM imaging and histological analysis have confirmed iron deposition within activated microglia and macrophages at the site of the paramagnetic rim (56). Subsequently, RNA sequencing identified two distinct microglia clusters in chronic active MS lesions: those involved in myelin phagocytosis and clearance, characterized by upregulation of genes involved in lipid metabolism, and those involved in antigen presentation, characterized by upregulation of genes such as the major histocompatibility complex (MHC) class II, ferritin complex, immunoglobulin Fc-γ receptors, and complement component C1 complex (35). Patients with at least one iron-rim lesion, as identified on QSM, have been shown to perform worse on physical and cognitive assessments than patients with no such lesions (58).

Paramagnetic rim lesions are found in approximately 50% of patients with RRMS and are speculated to indicate chronic neuroinflammation in MS (59, 60). These lesions are destructive, do not remyelinate, and tend not to shrink but expand over long periods of time, demyelinating surrounding tissue and promoting axonal loss (59–61). A different computational approach identifies slowly evolving/expanding lesions, which correlate with worsening Expanded Disability Status Scale (EDSS) scores and the development of SPMS in patients with RRMS (62). Thus, the measurement of both paramagnetic rim lesions and slowly evolving/expanding lesions may improve the specificity of MS diagnoses, and optimize assessments of patient prognosis (60).

Gadolinium-based contrast agents can be used to visualize the early stages of development of active lesions, when the blood-brain barrier temporarily becomes permeable, allowing the contrast agent to enter the CNS (63). The presence of a ring-like contrast enhancement and a peripheral hypointense rim on apparent diffusion coefficient (ADC) maps has been shown to predict the transition from acute to iron-rim lesions. However, the utility of gadolinium-based contrast agents is limited by the fact that immune cell infiltration into lesion areas occurs before the blood-brain barrier becomes permeable and continues after it closes (56).

The use of USPIOs provide insights into the early stages of the inflammatory process preceding gadolinium enhancement (64). USPIOs are administered intravenously and phagocytosed by peripheral monocytes. When these monocytes infiltrate the CNS, they can be visualized using T1-weighted imaging. A novel USPIO, Molday ION, has been shown to identify the location of microglia in the mouse model of MS, offering a new methodology which could potentially be used in human studies in the future (65).

PET provides functional imaging of the brain based on the uptake of radiolabeled tracers designed to target and bind to TSPO, which is upregulated on activated microglia and macrophages in early lesions (66, 67). ^11^C-PK11195 was the first radiotracer developed, and it has been widely used to study neuroinflammatory conditions (68). However, because of the limitations of ^11^C-PK11195, such as non-specific binding, newer radiotracers have been developed, including ^11^C-PBR28, ^18^F-DPA714, and ^18^F-FEPPA, that offer improved binding specificity and better signal-to-noise ratios (69, 70). Because TSPO is expressed on cells other than activated microglia, including certain subtypes of astrocytes and endothelial cells, the specificity of PET imaging remains suboptimal, even with second-generation TSPO-ligands (71). However, the reliability of PET imaging has been enhanced through meticulous mapping of TSPO-ligand binding to areas of interest in the MS brain, complemented by advanced post-processing techniques. This approach enables effective imaging with both the first-generation ligand ^11^C-PK11195 and the second-generation TSPO-ligands (72).

TSPO-PET imaging can be used to identify smoldering inflammation in NAWM, thalamus, and cortical gray matter in patients with MS (66). The distribution volume ratio (DVR) of TSPO-ligand binding is increased in patients with SPMS compared with patients with RRMS and healthy controls, indicating the presence of chronic inflammation (73, 74). Further, a study that used TSPO-PET imaging of patients at the presymptomatic stage of MS suggested that the choroid plexus may be a site of early inflammatory changes (including increased microglia infiltration), and that imaging of this brain region may represent a biomarker for early disease (75).

A method of automated TSPO-PET image analysis has been developed that can provide a quantitative assessment of microglial activation *in vivo* (76). A study using this method found that, compared with patients with RRMS, patients with SPMS had a higher proportion (19% vs 10%) and a higher median number (3 vs 1) of rim-active lesions, and that the median number of active voxels at the rim was higher in patients with SPMS than in patients with RRMS (158 vs 74). The number of active voxels at the rim and the volume of rim-active lesions were found to correlate with EDSS scores.

The detrimental effect of TSPO-PET-measurable microglial activation on axons is illustrated by the association between increased TSPO-binding and increased serum neurofilament light chain (NFL) levels (77). Moreover, a study using diffusion tensor imaging (DTI)-MRI showed a close correlation between microglial activation and microdamage in white matter tracts in the NAWM (74). Studies conducted in patients with MS have shown that TSPO-PET binding in the NAWM, in the thalamus, and at the rim of chronic lesions, indicating microglia- and macrophage-related pathology, is predictive of later disease progression independent of relapse activity (PIRA) (78–80).

Recent studies suggest that clusters of activated and proliferating microglia in the retina, visualized as hyperreflective foci using optical coherence tomography (OCT), are indicative of local inflammation and cortical pathology (81, 82). A study investigating the frequency of hyperreflective retinal foci and their association with retinal degeneration found an increased count of hyperreflective foci in patients with MS versus healthy controls (83). These data suggest that retinal microglia may be a useful biomarker for neurodegenerative diseases, and that OCT may represent a convenient, non-invasive, *in vivo* imaging modality to evaluate microglial activity in patients with MS.

## 7 Therapeutic targeting of microglia in MS

As a consequence of their involvement in the pathogenesis of MS, particularly as the predominant phagocytic cells in smoldering lesions and because of their involvement in PIRA, microglia represent an attractive potential therapeutic target (39). However, few of the drugs currently approved for the treatment of MS can influence the activity of microglia in a meaningful way, either because their mode of action does not target microglia, they cannot penetrate the blood-brain barrier, or because of inappropriate dosing regimens. Conversely, a number of drugs that are able to affect microglia activity are being investigated as potential treatments for MS, including BTK inhibitors, and also masitinib, ofatumumab, frexalimab, and rituximab.

BTK is a non-receptor tyrosine kinase expressed in a number of immune cell types, including B cells, monocytes, macrophages, mast cells, neutrophils, and microglia (84, 85). In addition, BTK is expressed in neurons and astrocytes (85). In microglia, BTK is involved in the activation and release of pro-inflammatory cytokines. Several BTK inhibitors are currently in the early stages of clinical development for the treatment of MS, including tolebrutinib, fenebrutinib, evobrutinib, remibrutinib, and orelabrutinib (**Table 2**).

Tolebrutinib is an oral, irreversible, selective BTK inhibitor that is able to enter the CNS at high rates (84). Tolebrutinib was evaluated in a randomized, placebo-controlled, phase 2b, dose-finding trial conducted in 130 patients with RMS or relapsing SPMS (86). In this trial, patients who were assigned to cohort 1 received tolebrutinib 5, 15, 30 or 60 mg/day for 12 weeks, followed by placebo for 4 weeks, while patients who were assigned to cohort 2 received placebo for 4 weeks, followed by tolebrutinib at the same doses for 12 weeks. The number of new gadolinium-enhancing lesions at week 12 was decreased in a dose-dependent manner, as patients who received higher doses of tolebrutinib had fewer lesions. The most common adverse events associated with tolebrutinib were headache, upper respiratory tract infection, and nasopharyngitis. In the long-term extension study, approximately 81% of patients remained relapse-free after 2 years (87). Several phase 3 clinical trials of tolebrutinib are ongoing (**Table 2**).

Evobrutinib is another oral, irreversible, selective BTK inhibitor (84). Evobrutinib, at doses of 25 mg or 75 mg once daily or 75 mg twice daily, was evaluated in a randomized, double-blind, placebo-controlled, phase 2 trial conducted in 267 patients with RMS or SPMS with superimposed relapses (88). Patients who received evobrutinib 75 mg once daily had significantly fewer gadolinium-enhancing lesions at weeks 12 to 24 than patients who received placebo. There were no significant differences in the number of gadolinium-enhancing lesions between patients who received other doses of evobrutinib or those who received placebo. In addition, there were no significant differences in the annualized relapse rate or disability progression between patients who received evobrutinib at any dose and those who received placebo. However, it should be noted that the study was not powered to assess these endpoints. The most common adverse events associated with evobrutinib were nasopharyngitis and increased levels of liver enzymes. In an open-label extension of this study, all patients were switched to evobrutinib 75 mg once daily at week 48, then moved to evobrutinib 75 mg twice daily after additional analyses demonstrated the superiority of twice-daily dosing for several clinical, imaging, and biomarker endpoints (89). This open-label extension showed that the efficacy of evobrutinib 75 mg twice daily during the double-blind period was maintained up to week 192. A *post hoc* analysis of this study additionally showed that treatment with evobrutinib resulted in a decrease in the volume of slowly expanding lesions relative to placebo, particularly in patients who received 75 mg twice daily (90). Unfortunately, preliminary results from two phase 3 clinical trials of evobrutinib 45 mg twice daily in patients with RMS (evolutionRMS 1 and evolutionRMS 2; **Table 2**) found that, owing to lower than expected annualized relapse rates in patients randomized to oral teriflunomide, evobrutinib failed to meet the primary endpoint of reducing annualized relapse rates (91). A number of clinical trials of fenebrutinib, remibrutinib, and orelabrutinib are also ongoing (**Table 2**) (84).

In addition to BTK inhibitors, other novel drugs are being evaluated in the treatment of MS (**Table 2**). Masitinib is an orally administered tyrosine kinase inhibitor (92). Masitinib, at doses of 4.5 mg/kg/day or 6.0 mg/kg/day, was evaluated in a randomized, placebo-controlled trial conducted in 611 patients with PPMS or nonactive SPMS (93). Patients who received masitinib 4.5 mg/kg/day had significantly smaller changes in the EDSS score from baseline to week 96 than patients who received placebo, indicating a slowing of disease progression. However, no statistically significant differences were observed in the change in the EDSS score between patients who received masitinib 6.0 mg/kg/day and those who received placebo.

Ofatumumab is a human anti-CD20 monoclonal antibody (94). With specific emphasis on microglia, ofatumumab was evaluated in an open-label, observational study of 10 patients with relapsing forms of MS. An interim analysis showed that ofatumumab treatment was associated with decreased activation of microglia in cortical gray matter, assessed using ^18^F-PBR06 PET scans. In addition, depletion of peripheral CD19-positive cells was observed at day 5, which suggests that B cells may influence microglial activity in patients with RMS.

Frexalimab, a CD40L inhibitor capable of influencing both innate and adaptive immune responses, was evaluated for the treatment of RMS in a phase 2 trial (95). The trial involved a 12-week double-blind, randomized, placebo-controlled period (part A), followed by an ongoing open-label extension (part B). Participants were assigned to either high-dose frexalimab, low-dose frexalimab, high-dose placebo, or low-dose placebo groups. At week 12, high-dose frexalimab was associated with an 89% reduction in new gadolinium-enhancing T1 lesions compared with pooled placebo, and low-dose frexalimab was associated with a 79% reduction in these lesions. Both high and low doses of frexalimab demonstrated significant reductions in new/enlarging T2 lesions and total number of gadolinium-enhancing T1 lesions at week 12. In addition, 85% of participants in the high-dose group and 84% in the low-dose group were free of new gadolinium-enhancing T1 lesions at week 12. The Multiple Sclerosis Impact Scale 29 (MSIS-29) physical impact score significantly improved at week 12 with high-dose frexalimab compared with placebo. Additionally, high-dose frexalimab led to a 24% reduction, and low-dose to a 18% reduction, in plasma NFL levels at week 12 versus pooled placebo. Frexalimab was well tolerated, with no serious or severe treatment-emergent adverse events reported during the double-blind period. The most common adverse events were COVID-19 and headache. Phase 3 trials of frexalimab in MS are ongoing in 2024 (**Table 2**).

Lastly, an *in vivo* TSPO-PET imaging study found that natalizumab, an α_4_-integrin antagonist, reduced microglia activation over 1 year of treatment in 10 patients with SPMS or RRMS (96). Treatment with rituximab, a monoclonal antibody against CD20, over an 18-month period similarly resulted in a reduction in TSPO-PET binding in a patient newly diagnosed with PPMS (97).

## 8 Conclusions and future directions

Microglia play a wide range of roles in CNS development and immunological surveillance. As further information emerges regarding the role of microglia in the pathogenesis of MS, this may help to advance the clinical development of novel treatments for MS patients, particularly for those with progressive disease. Further research on the role of microglia in MS and clinical trials of drugs targeting microglia, either directly (e.g., BTK inhibitors) or indirectly (e.g., frexalimab), may improve outcomes in patients with this debilitating condition. Future studies should also evaluate the effect of targeting microglia on outcomes that better reflect the progressive nature of MS (e.g., patient-reported outcome measures, composite scores), utilize imaging modalities such as PET to select patients and monitor microglial activity, and include provisions to extend study durations according to prespecified time-to-event plans. These advances in MS research have the potential to improve the conduct and findings of clinical trials, and provide new treatment strategies for the long-term management of patients with MS.
